# Effect of Sugar-Free Jelly on Glycemic Metabolism and Its Potential Health Benefits in Non-Diabetic Adults

**DOI:** 10.3390/foods13060920

**Published:** 2024-03-18

**Authors:** Heejin Han, Yuri Kim, Minchul Gim, Hoyeon Shin, Hyunsook Jang, Won Joo Yoon, Gyeong-Hweon Lee, Yoo Kyoung Park

**Affiliations:** 1 Department of Medical Nutrition, Graduate School of East-West Medical Science, Kyung Hee University, Yongin 17104, Republic of Korea; k2022510049@khu.ac.kr (H.H.); uri0516@khu.ac.kr (Y.K.); 2 Lotte R&D Center, Seoul 07594, Republic of Korea; minchul.gim@lotte.net (M.G.); hoyeon.shin@lotte.net (H.S.); hyunsook.jang@lotte.net (H.J.); wjyoon@lotte.net (W.J.Y.); ghlee1@lotte.net (G.-H.L.); 3 Department of Medical Nutrition, AgeTech-Service Convergence Major, Graduate School of East-West Medical Science, Kyung Hee University, Yongin 17104, Republic of Korea

**Keywords:** sugar-free, sugar alcohols, polyol, glucose metabolism, glycemic control, appetite

## Abstract

Excessive sugar consumption provides energy but has little nutritional value, contributing to the prevalence of obesity. Hence, “sugar-free” products using artificial or natural sweeteners, including sugar alcohols, have become popular. Accordingly, safety concerns and curiosity have arisen. Therefore, this study used a double-blind, crossover design to compare the effects of commercial sugar-free and sugar jellies (control) on the glycemic response in 16 adults without diabetes. Blood samples were collected to measure blood glucose, insulin, glucagon, ghrelin, C-peptide, glycated hemoglobin, and glycated albumin levels, and an oral glucose tolerance test was performed. Questionnaires on satiety and intestinal health were also administered. Sugar-free jellies resulted in significantly lower glucose and insulin levels and a reduced area under the curve while showing higher glucagon levels than the controls. Moreover, the sugar-free jelly initially resulted in the greater secretion of ghrelin; however, after 2 h, the control jelly resulted in higher ghrelin. No significant differences were observed in gut quotient, C-peptide, glycated hemoglobin, and glycated albumin levels. In conclusion, substituting sugar jelly with sugar-free jelly may induce lower blood glucose and insulin levels and higher glucagon levels, indicating a better ability to control glucose metabolism. Appetite was not stimulated by sugar-free jelly consumption.

## 1. Introduction

Excessive sugar consumption provides sufficient energy but offers little nutritional value and can lead to obesity [1]. Moreover, it causes many health problems such as hypertension [2] and the potential risk of cancer [3]. For instance, the consumption of more than 12 oz of sugar-sweetened beverages was associated with a 12% increase in the risk of hypertension in both men and women [2]. Furthermore, people who consumed 135 g of sugar a had 17% higher risk of all cancers than people who consumed 55 g of sugar according to a web-based cohort in France [3]. In an effort to reduce the health risks associated with excessive sugar intake, the World Health Organization (WHO) recommends limiting sugar intake to less than 10% of the total energy intake [1]. Accordingly, the European Union mandates the inclusion of signs of total sugar content on food labels, imposes taxes on sugar-containing products such as beverages and ice creams, and encourages the use of sugar alternatives in food production [1].

Alternative sugars can decrease the total sugar and overall energy intake in the population, eventually contributing to a lower incidence of obesity and type 2 diabetes [4]. Thus, it is crucial to make sugar-free products more accessible and satisfy customer expectations regarding taste and price. A positive consumer perspective on taste has been previously demonstrated; a study on reduced-sugar cookies found that consumers preferred a less sweet taste and were willing to pay up to 10% more for sugar-reduced products [5]. Numerous other studies have explored sugar reduction, including the quality characteristics of reduced-sugar cookies [6], quality attributes in cookies using sugar alcohols [7], and the use of specific ingredients to lower sugar content [8]. Moreover, one study demonstrated that the use of alternative sugars can lead to reduced blood glucose levels compared with the use of sugar [9]. Despite these findings, the WHO advised against the use of non-sugar sweeteners (NNSs) as a method to lose weight or prevent non-communicable diseases [10]. This advice was based on a review of 283 studies that led to confusion between consumers and industries because the WHO recommended using NNSs to reduce sugar consumption [10].

The NNSs mentioned in the WHO recommendation encompass a range of natural or modified sweeteners, including acesulfame K, aspartame, advantame, cyclamate, neotame, saccharin, sucralose, stevia, and stevia derivatives [10]. In addition to these NNSs, sugar alcohols, also known as polyols, such as sorbitol, maltitol, erythritol, and xylitol, are not considered sugars and may be suitable alternatives for sugar reduction in food products [11]. Sugar alcohols have a lower influence on blood glucose and insulin responses, taste similar to sugar, and have a lower calorie content as they are not fully digested and absorbed by the body; these advantages make them a suitable choice for individuals who struggle to control their blood glucose levels [12,13]. Nevertheless, owing to their indigestibility, consuming excessive amounts of sugar alcohols may lead to various digestive symptoms, such as bloating and abdominal discomfort, depending on an individual’s gastrointestinal health [11,13]. As no specified acceptable daily intake (ADI) of sugar alcohols has been established, caution is necessary when adding them to the diet [13].

Research findings suggest that artificial sweeteners may increase appetite [14] and may not have the expected benefits on body weight or glycemic regulation [15,16]. Nevertheless, most studies on sugar-free products focused on their sensory qualities and overall characteristics. Considering these health concerns, we aimed to examine the acute effects of consuming sugar-free products on blood glucose levels and appetite in healthy adults.

## 2. Materials and Methods

### 2.1. Recruitment and Population

Sixteen participants were recruited from the general public through posters and advertisements placed around the hospital in Seoul, Korea. The inclusion criteria were as follows: adults without diabetes, aged between 19 and 65 years, with fasting blood glucose levels below 125 mg/dL, and who voluntarily signed up for the study. Individuals were excluded if they were pregnant or lactating; had used hypoglycemic agents in the 3 months preceding the study; had any medical conditions, including thyroid and gastrointestinal disorders; weighed less than 40 kg; had anemia; had metabolic syndrome; were currently on medications or treatments for other illnesses; engaged in heavy alcohol consumption more than four times a week; lacked the innate ability to break down glycolytic fermentation; had high blood pressure; or were concurrently participating in another clinical trial or planning to do so at the initial visit.

According to the International Organization for Standardization (ISO), at least 10 people are required to test glycemic indexes (GIs) [17]. However, to accurately determine the required number of participants for this study, we referred to the most similar study exploring the impact of consuming low-calorie muffins containing maltitol and high-amylose corn starch on postprandial response and satiety [18]. In this study, which included 14 participants, statistically significant postprandial glycemic and insulinemic responses were observed, and there were no dropouts. Considering a potential 10% dropout rate associated with the crossover design, we decided on a total sample size of 16 participants.

### 2.2. Experimental Design

This study was a clinical trial. The participants were divided into two groups, and each group consumed two products (sugar-free and sugar jelly [control]) alternatively. Identification numbers were allocated by an unrelated party and used to distribute products to participants during the study period.

**Figure 1:**
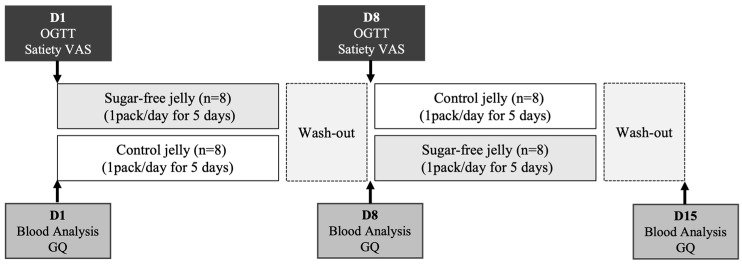
Study design.

An oral glucose tolerance test (OGTT) was performed over a 2 h period following a test meal to analyze postprandial responses, including glucose, insulin, glucagon, and ghrelin levels. Before consuming the test products, c-peptide, glycated hemoglobin (HbA1c), and glycated albumin (GA) levels were measured. Furthermore, participants completed two surveys to measure their satiety and intestinal health. Following the initial day of product consumption, participants consumed either sugar-free or control jellies after lunch for the following 4 days to assess their effects on intestinal activity. The participants were instructed to consume one pack of jelly during lunch time, and lunch was not provided. After a 2-day washout period, they switched to another product that they had not previously consumed. During the washout period, participants were instructed to avoid high-sugar products and alcohol while maintaining similar eating habits (Figure 1). This study was approved by the ethics committee of the Kyung Hee University with the approval number KHGIRB-23-159. Informed consent was obtained from all subjects involved in the study. 

### 2.3. Products

**Figure 2:**
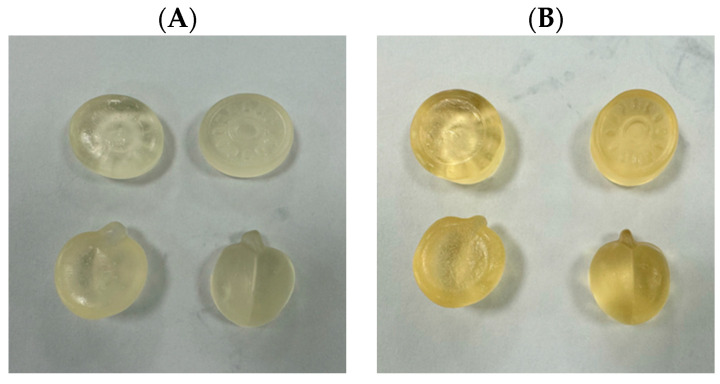
Products being used. (**A**) Sugar-free jellies; (**B**) control jellies.

Commercially available sugar-free products, sourced from LOTTE Wellfood Co., Ltd. (Seoul, Republic of Korea), were formulated to replace sugar with sugar alcohols. In contrast, control samples were produced for research purposes. The sugar-free jelly contained 30.7 g of maltitol syrup, whereas the control jelly contained 31.5 g of sugar. The products are shown in Figure 2.

The GI serves as a metric for assessing the effect of carbohydrates on blood sugar levels, typically expressed as the area under the blood sugar curve for 2 h after consuming 50 g of carbohydrates in 10 people. According to ISO standards, GI tests require 50 g or 25 g of glycemic carbohydrates [17]. However, these particular carbohydrate amounts were unsuitable owing to the possibility of gastrointestinal discomfort associated with low-digestible carbohydrates, such as resistant starch and sugar alcohols. To meet these standards, a minimum of 10 g of glycemic carbohydrates per serving was required. Therefore, the test jellies contained 30 g of glycemic carbohydrates per serving.

The participants consumed one serving, equivalent to 52 g of jelly (1 pack, 16–17 pieces) within 10 min without water. The nutritional values of the products are listed in Table 1.

### 2.4. Blood Analysis

Participants arrived at the hospital after a 12 h fasting period. An intravascular catheter was then placed in each arm and blood samples were collected for analysis. In this study, three biomarkers were used to evaluate the impact on glycemic metabolism: C-peptide was used to assess insulin secretion [19], and HbA1c and GA were used to determine the level of glycation. Because HbA1c is calculated as the ratio of glycated hemoglobin to total hemoglobin, it is affected by the lifespan and glucose levels of erythrocytes. As a better biomarker that is unaffected by erythrocytes, GA is increasingly used as an alternative to HbA1c [20,21].

On the first day of consuming both types of jelly, the participants underwent an OGTT after consuming the jellies. Blood samples were collected at specific time intervals (0, 15, 30, 60, 90, and 120 min after consumption) to monitor blood glucose and insulin levels. For glucagon and ghrelin, blood samples were collected 0, 90, and 120 min after consumption. C-peptide, HbA1c, and GA levels were measured before consumption on day 1, day 8, and day 15. The results from day 1 were used as the baseline. Subsequently, all blood samples were centrifuged, and the serum for ghrelin was stored at −20 °C for further analysis. 

### 2.5. Satiety

Participants were asked to assess their subjective appetite using a 100 mm visual analogue scale (VAS) both before consumption and at 30, 60, and 120 min post-consumption [22]. Satiety was evaluated on the same day as OGTT. The appetite evaluation encompassed eight questions: how hungry are you? (not hungry at all: 0 mm, intolerably hungry: 100 mm); how satisfied do you feel? (completely empty: 0 mm; cannot eat anything: 100 mm); how full do you feel? (not full at all: 0 mm, excessively full: 100 mm); how much do you think you can eat? (cannot eat anything: 0 mm; capable of eating a lot: 100 mm); would you like to eat something sweet? (strongly desire to eat: 0 mm; not at all: 100 mm); would you like to eat something salty? (strongly desire to eat: 0 mm, not at all: 100 mm); would you like to eat something savory? (strongly desire to eat: 0 mm; not at all: 100 mm); and would you like to eat something fatty? (strongly desire to eat: 0 mm; not at all: 100 mm). Hence, higher scores indicated more severe hunger, whereas lower scores indicated a stronger desire to eat specific foods. 

### 2.6. Gut Quotient

The excessive consumption of sugar alcohols can lead to bowel discomfort, diarrhea, or abdominal pain [11]. To evaluate intestinal health, the Korean version of gut quotient (GQ) measurement scale [23] was used. The GQ comprises 17 questions categorized into 3 factors: perceived intestinal discomfort, bowel movement discomfort, and bowel movement control discomfort. The importance of each question is reflected in its score, which is then multiplied to calculate the final GQ score. The highest score on this scale is 100. 

The GQ scale was administered at three time points: on the first day, after 1 week, and at the end of the study. The initial GQ score on the first day served as the baseline for comparison with GQ scores after 1 week and on the final day, and differences from baseline measurements helped evaluate changes in bowel condition over the course of the study.

### 2.7. Statistical Analysis

IBM SPSS Statistics for Macintosh, Version 28.0 (Armonk, NY, USA: IBM Corp), was used for all analyses, and the level of statistical significance was set at a *p*-value < 0.05 in a two-sided test. The Shapiro–Wilk test was used to assess normality at a 5% significance level. Differences between the sugar-free and control groups were evaluated using the independent *t*-test when the *p*-value was >0.05 and the Mann–Whitney U test when the *p*-value was <0.05. The results are presented as means and standard deviations. The areas under the curves (AUCs) were calculated for each variable using the trapezoidal method in Microsoft Excel.

## 3. Results

### 3.1. General Participant Characteristics 

A total of 16 participants were included in this study. The average body mass index (BMI) slightly exceeded the normal range, and the mean fasting glucose levels indicated that the participants were not in the diabetic range. The average systolic and diastolic blood pressure and pulse rate were within the normal range. To confirm diabetes status, C-peptide, HbA1c, and GA levels were assessed, and all diabetes biomarkers were within the normal range. The general patient characteristics are shown in Table 2. No participants withdrew; therefore, data from 16 participants who completed the 2-week study period were included in the analysis.

### 3.2. Glucose Metabolism Associated Biomarkers

**Figure 3:**
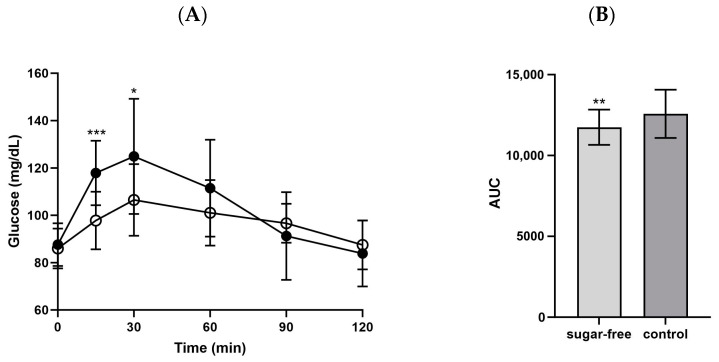
(**A**) Glucose changes and (**B**) AUC 120 min after consuming the jellies. ○: sugar-free jelly; ●: control jelly. Significant differences at *p* < 0.05 compared using the independent *t*-test. * *p* < 0.05, ** *p* < 0.01, *** *p* < 0.001. Abbreviations: AUC: area under the curve.

The mean blood glucose level at 15 min post-consumption of the sugar-free jelly was 97.8 ± 12.2 mg/dL, whereas that of the control jelly was 117.9 ± 13.7 mg/dL (*p* = 0.0001). After 30 min, the sugar-free jelly resulted in significantly lower blood glucose levels (106.5 ± 15.1 mg/dL) compared with the control jelly (124.9 ± 24.3 mg/dL; *p* = 0.016, Figure 3). 

**Figure 4:**
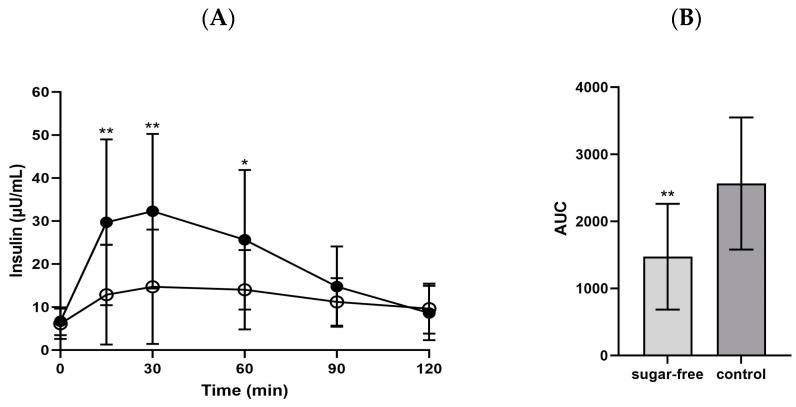
(**A**) Insulin changes and (**B**) AUC 120 min after consuming the jellies. ○: sugar-free jelly; ●: control jelly. Significant differences at *p* < 0.05 compared using the independent *t*-test. * *p* < 0.05, ** *p* < 0.01. Abbreviations: AUC: area under the curve.

Moreover, the insulin level at 15 min after sugar-free jelly consumption (97.8 ± 12.2 µU/mL) was significantly lower than that after control jelly consumption (117.9 ± 13.7 µU/mL; *p* = 0.004). This trend continued at 30 and 60 min post-consumption, with the sugar-free jelly resulting in insulin levels of 14.7 ± 13.3 µU/mL and 14.0 ± 9.2 µU/mL compared with the control jelly resulting in insulin levels of 32.3 ± 18.0 µU/mL and 25.6 ± 16.3 µU/mL, respectively. The insulin AUC after consuming the sugar-free jelly was significantly lower compared with that after consuming the control jelly (*p* = 0.004, Figure 4).

**Figure 5:**
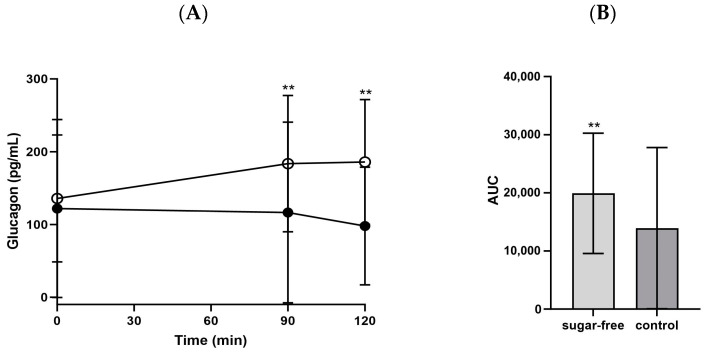
(**A**) Glucagon changes and (**B**) AUC 120 min after consuming the jellies. ○: sugar-free jelly; ●: control jelly. Significant differences at ** *p* < 0.05 compared using the independent *t*-test. Abbreviations: AUC: area under the curve.

Glucagon exhibited an opposite trend to that of insulin because of its contrary function. Significantly higher increases in glucagon levels were observed at 90 and 120 min post-consumption of the sugar-free jelly compared with those post-consumption of the control jelly (90 min: 183.7 ± 93.6 pg/mL vs. 116.5 ± 124.2 pg/mL, *p* = 0.001; 120 min: 185.9 ± 85.7 pg/mL vs. 98.0 ± 80.8 pg/mL, *p* = 0.002; Figure 5). Additionally, the AUC for glucagon was significantly higher after sugar-free jelly consumption (332.0 ± 172.5) compared with after control jelly consumption (232.4 ± 232.3, *p* = 0.003), which is intimately linked to the trends observed in insulin levels.

Regarding the three biomarkers, C-peptide, HbA1c, and GA, used to evaluate the impact of the jellies on glycemic metabolism, no significant differences were observed among the sugar-free jelly, control jelly, and baseline, as shown in Table 3.

### 3.3. Satiety and Ghrelin

**Figure 6:**
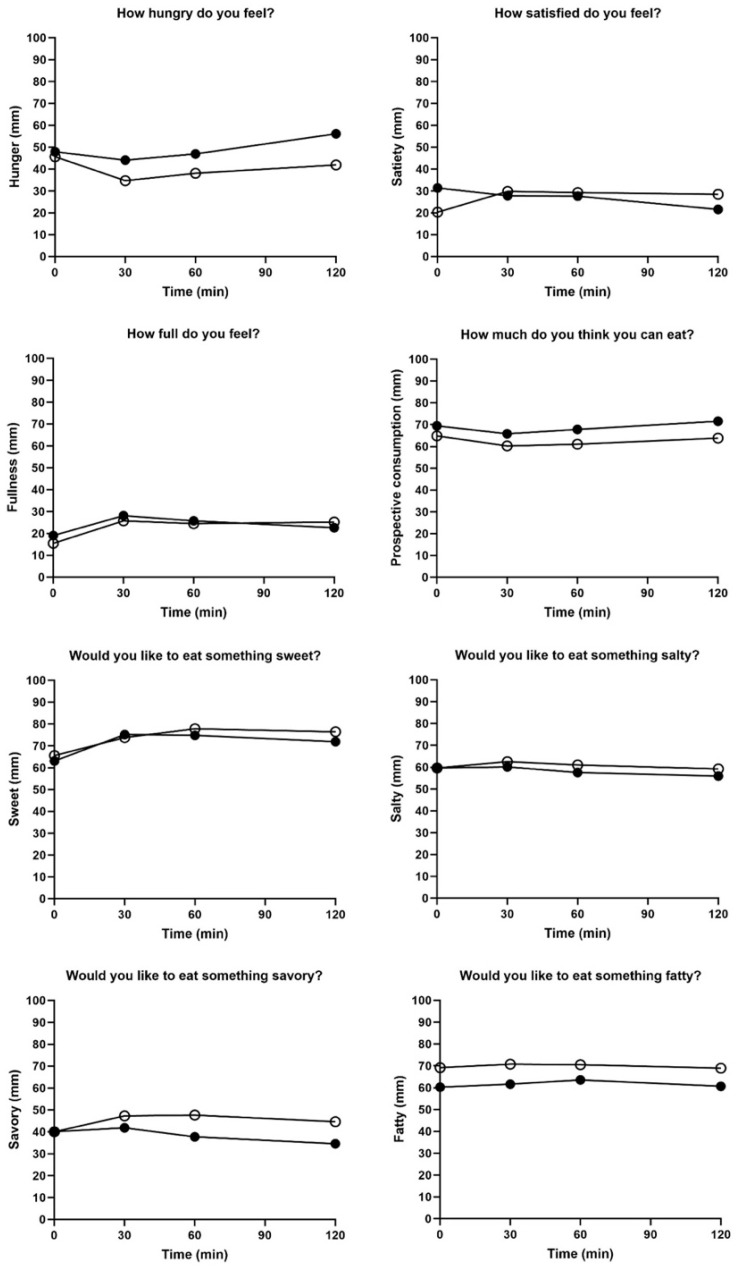
Subjective appetite scores after eating the jellies. ○: sugar-free jelly; ●: control jelly. No significant differences at *p* < 0.05 by non-parametric test (Mann–Whitney test).

Figure 6 shows the subjective analysis of satiety after consuming the jellies; no significant differences were observed in appetite scores. Although no significant differences in ghrelin levels were observed over 120 min, unusual ghrelin-related findings were observed. Specifically, the change in ghrelin values from 0 to 90 min was significantly lower after sugar-free jelly consumption than after control jelly consumption. However, between 90 and 120 min, the sugar-free jelly resulted in a slight decrease in ghrelin levels, whereas the control jelly resulted in an increase. This indicated that the sugar-free jelly initially led to the greater secretion of ghrelin, a hormone associated with appetite. After 2 h, ghrelin secretion was higher in the control jelly group than in the sugar-free jelly group (Table 4).

### 3.4. GQ

The baseline GQ score was 85.75 ± 15.15. After 5 days of sugar-free jelly consumption, the GQ decreased to 72.31 ± 25.62, whereas the GQ score after consuming control jelly was 87.81 ± 10.50 (Table 5). Although some participants reported discomfort during sugar-free jelly consumption, no statistically significant differences in GQ scores were observed between the sugar-free and control jelly groups (*p* = 0.186).

## 4. Discussion

This study attempted to assess the postprandial glycemic and insulinemic responses, as well as the effects on satiety and gastrointestinal symptoms, associated with sugar-free jelly containing sugar alcohols in comparison to a control jelly containing sugar.

The primary finding of this study was the potential of sugar-free jellies containing sugar alcohols to positively influence glycemic metabolism. Specifically, the consumption of sugar-free jelly led to lower postprandial glucose levels. These findings are supported by a prior study comparing regular strawberry jelly to jelly for those with diabetes with sucralose and dextrin, demonstrating that reduced-sugar jelly consumption with an identical meal did not spike glucose and insulin levels in type 2 diabetes under controlled conditions [24]. Given that the current study did not provide the same meal and involved participants without diabetes, the observed effect of sugar-reduced jelly on glucose levels is understandable.

The second important finding of our study was regarding the responses to insulin and glucagon. Insulin, secreted from β cells, acts to decrease blood glucose levels in response to their rise, mirroring the trend of glucose. A study by Thabuis et al. showed that consuming 150 mL of water with 50 g of maltitol, a type of polyol, resulted in lower insulin levels compared with the same amount of water with the same amount of glucose, consistent with the findings in this study [25]. Insulin plays a crucial role in reducing blood glucose levels by inhibiting glucose production in the liver and increasing glucose absorption by muscles and fat. Excessive insulin secretion inhibits fat breakdown and promotes pre-adipocyte differentiation, ultimately increasing the risk of obesity [26]. In this study, the total insulin AUC after consuming sugar-free jellies was lower than that after consuming the control jellies, indicating that sugar-free products utilizing sugar alcohols led to reduced insulin variability compared with control jellies using sugar. These results suggest that sugar-free products are promising alternatives for individuals with impaired insulin regulation. This implies that sugar-free products may be a better choice for maintaining normal blood glucose levels.

The consumption of carbohydrates, particularly sugar, can stimulate appetite and increase food intake [27]. Considering that sugar-free jellies lack sugar, we hypothesized that the sensation of satiety might decrease while appetite increases after sugar-free jelly consumption compared with control jelly consumption. However, the third finding of this study revealed that sugar-free jelly consumption resulted in no significant differences compared with the control jelly with regard to ghrelin values and satiety measured using the VAS scale. This result is in line with the results of a study in 2010 involving 31 individuals, conducted over 3 days, which examined the impact of snacks with aspartame, stevia, and sugar before a meal and found that consuming snacks comprising tea, crackers, and cream cheese using low-calorie alternative sweeteners did not affect subsequent meal quantity and satiety [9]. This finding implies that the use of alternative sugars may not reduce satiety or increase energy intake. In contrast, a comparison between water containing 240 mg of saccharin and 75 g of sugar indicated that drinking saccharin water resulted in higher ghrelin levels and a greater desire to eat than sugar-sweetened water [14]. The distinction between these studies was based on the form of consumption, with one representing “food”, while the other was a “drink”. Notably, the “food” contained additional ingredients beyond sweeteners, presenting a challenge in isolating the effects attributed to sweeteners.

Intriguingly, ghrelin levels at specific time points showed no significant differences, whereas the percentage change values from 0 to 90 min and from 90 to 120 min were significantly different. This study revealed that sugar-free jelly initially induced greater ghrelin secretion; however, after 120 min, the control jelly group exhibited higher ghrelin secretion than the sugar-free jelly group. The sugar in the control jelly caused rapid fluctuations in blood sugar, prompting the body to find energy to maintain homeostasis, thus increasing appetite-promoting hormones. 

Despite these discoveries, there are several limitations. Primarily, despite efforts to encompass a diverse age demographic, individuals aged 53 and above were not enlisted, thereby impeding the representativeness of the study. Second, the study was conducted to explore the potential of sugar-free jelly as a suitable snack for diabetic patients or individuals at risk of chronic diseases needing blood sugar management. However, as it was actually conducted on healthy individuals, it may not be applicable to diabetic patients. The strength of this study, however, is that the products used were confectionaries with 100% of their sugar removed without sacrificing sensory acceptance. Previous studies have used reduced-sugar products, often utilizing jellies with a minimal sugar alternative rather than a complete substitution of sugar with sugar alcohols. These include studies on quality characteristics [6,28], the evaluation of attributes in jellies using sugar alcohols [7], and the examination of specific ingredients to lower sugar content [8]. To the best of our knowledge, this study marks the first evaluation of market-available sugar-free products in adults without diabetes. In addition, our crossover design minimized intra-variability, enhancing the effectiveness of exploring the impact of sweetener variations and providing clinical significance to the observed statistical differences.

## 5. Conclusions

The consumption of sugar-free jellies displayed preferable effects on glucose metabolism, especially in reducing glucose and insulin levels while increasing glucagon levels, compared with sugar-containing control jellies. Although the participants did not have diabetes, these findings suggest the potential benefits of managing postprandial glucose and insulin levels. Contrary to our initial hypothesis, the consumption of sugar-free jelly did not promote appetite or food consumption. These results clearly indicate that the consumption of sugar-free products has a lower impact on glycemic metabolism, accompanied by a reduction in appetite, than sugar-containing products.

## Figures and Tables

**Table 1:** Nutritional values of jellies.

	Sugar-Free	Control
Calorie (kcal)	126.5	187.0
Carbohydrate (g)	40.2	42.3
Sugar (g)	0.0	31.5
Sugar alcohols (g)	30.7	0.0
Protein (g)	3.4	3.8
Fat (g)	0.1	0.3

**Table 2:** Anthropometric and clinical characteristics of the participants.

	*n* = 16	Reference Values
Age (years) [Range]	35.8 ± 7.5 [26–52] ^1^	-
Male/female (*n*)	3/13	-
Height (cm)	165.3 ± 7.1	-
Weight (kg)	63.4 ± 12.9	-
Fasting glucose (mg/dL)	107.9 ± 9.2	70–99
BMI (kg/m^2^)	23.1 ± 3.9	18.5–22.9
Systolic blood pressure (mmHg)	116.8 ± 14.1	<120
Diastolic blood pressure (mmHg)	74.3 ± 13.1	<80
Pulse rate (bpm)	77.6 ± 8.9	60–100
C-peptide (ng/mL)	2.1 ± 0.5	1.10–4.40
HbA1c (%)	5.3 ± 0.3	<5.7
Glycated Albumin (%)	12.3 ± 1.2	11–16

BMI: body mass index. ^1^ Data are presented as means ± standard deviations.

**Table 3:** C-peptide, HbA1c, and GA values at baseline and after consuming jellies.

	Baseline	Sugar-Free	Control	*p*-Value ^2^
C-peptide (ng/mL)	2.06 ± 0.53 ^1^	1.88 ± 0.50	1.79 ± 0.49	0.418
HbA1c (%)	5.31 ± 0.29	5.26 ± 0.27	5.23 ± 0.29	0.819
GA (%)	12.26 ± 1.19	12.52 ± 0.95	12.58 ± 0.90	0.792

^1^ Data are presented as means ± standard deviations. ^2^ No significant differences at *p* < 0.05 as analyzed using non-parametric test (Mann–Whitney test). Abbreviations: HbA1c, glycated hemoglobin; GA, glycated albumin.

**Table 4:** Ghrelin values and changes over 120 min.

	Sugar-Free	Control	*p*-Value ^2^
Ghrelin (pg/mL)			
0 min	1368.7 ± 489.6 ^1^	1303.1 ± 488.3	0.462
90 min	1197.3 ± 329.7	1078.6 ± 389.9	0.142
120 min	1162.6 ± 258.6	1185.8 ± 439.9	0.763
% change			
90 min/0 min	−9.3 ± 12.8	−16.5 ± 9.8	0.042 *
120 min/90 min	−1.5 ± 10.2	9.7 ± 8.0	0.002 **

^1^ Data are presented as means ± standard deviations. ^2^ Significant differences (*p* < 0.05) as analyzed using non-parametric test (Mann–Whitney test). * *p* < 0.05, ** *p* < 0.01.

**Table 5:** Gut quotient (GQ) at baseline and after 5 days of jelly consumption.

	Baseline	Sugar-Free	Control	*p*-Value ^2^
GQ	85.75 ± 15.15 ^1^	72.31 ± 25.62	87.81 ± 10.50	0.186

^1^ Data are presented as mean ± standard deviations. ^2^ No significant differences at *p* < 0.05 by non-parametric test (Mann–Whitney test).

## Data Availability

The original contributions presented in the study are included in the article, further inquiries can be directed to the corresponding author.
